# Developmental program impacts phenological plasticity of spring wheat under drought

**DOI:** 10.1186/s40529-016-0149-3

**Published:** 2016-11-03

**Authors:** Marwa N. M. E. Sanad, Kimberley Garland Campbell, Kulvinder S. Gill

**Affiliations:** 1 Crop and Soil Sciences Department, Washington State University, Pullman, WA USA; 2 Genetics and Cytology Department, National Research Center, Giza, Egypt; 3 Wheat Genetics, Quality, Physiology, Diseases Research, USDA-ARS, Pullman, WA USA

**Keywords:** Phenological plasticity, Developmental plasticity, Tillering stage, Drought, Spring wheat

## Abstract

**Background:**

Developing drought-tolerant crops critically depends on the efficient response of a genotype to the limited water availability, a trait known as phenological plasticity. Our understanding of the phenological plasticity remains limited, in particular, about its relationships with plant developmental program. Here, we examined the plastic response of spring wheat at tillering, booting, heading, and anthesis stages to constant or periodic drought stress. The response was assessed by morphological and physiological parameters including symptoms.

**Results:**

The dynamics of morphological symptoms were indicators of the plasticity identification of drought. We found that spring wheat exhibits higher phenological plasticity during tillering stage followed by the heading stage, while booting and anthesis stages are the most sensitive. Also, the adaptive response is thought to be influenced with the plant height genes. Furthermore, periodic stress caused more pronounced inhibition of yield than the constant stress, with limited resistance resolution under long period.

**Conclusions:**

Our study shows the importance of considering the phenological plasticity in designing screens for drought tolerance in spring wheat and proposes tillering as the most informative stage for capturing genotypes with tolerance to limit water availability.

## Background

Low precipitation is a serious obstacle to sustainable farming. The negative impact of drought on the quality and quantity of major crop such as wheat *(Triticum aestivum* L.) yields threatens global food security. Several strategies are used by plant species to achieve drought tolerance including escape, avoidance, and resilience also known as phenotypic plasticity; (Farooq et al. 2009). However, the phenotypic plasticity can be limited by several environmental factors (Nicotra and Davidson 2010). Some of these factors are: the developmental plasticity, phenological plasticity (a predisposition for phenotypic plasticity), or length of the stress period.

Initially, phenotypic responses can result in an acclimation to drought. Continuing water deficit can then lead to more stable tolerance through adaptation mechanisms (adaptive plasticity) (Nicotra and Davidson 2010). The frequency and duration of the drought episodes play a major role in defining the degree of tolerance. The accumulated history of adaptive plasticity events generates a stresses imprint that can be revoked during the subsequent stress episodes. This phenomenon was defined as “plant memory” (Bruce et al. 2007; Aubin-Horth and Renn 2009; Walter et al. 2011) replacing previous term “immunological memory” used to explain damage-induced signaling (Baldwin and Schmelz 1996).

Plant evolution and plant ecology are determined to be influenced by the developmental plasticity, which contribute to improving yield stability in agriculture (de Jong and Leyser, 2012). Because the efficiency of the phenotypic plasticity is determined by the developmental stage, stress duration and stress severity in the genotype-dependent manner (Sivamani et al. 2000; Chaves et al. 2003; Rizza et al. 2004; Milad et al. 2011). Thus, apart from defining the stress protocol, screening for drought tolerance requires a careful consideration of the growth stage at which the tolerance will be scored. Three consequences of phenotypic plasticity as a drought response were observed in plants: 1) phenotypic changes after single stress exposure (Noormets et al. 2008), 2) retrieving the positive phenotype after recurrent stress exposure (Bruce et al. 2007), and 3) overcoming rewatering stress (Xu et al. 2010). Successive drought and rewatering cycles can shift the time frame of plant development or phenological plasticity (Vitasse et al. 2010), and change the final biomass or leaf area (Xu et al. 2010). These factors highlight the importance of assessing the phenological plasticity for capturing the drought tolerant genotypes.

Development of “plant memory” for response to drought is determined by the time interval between consecutive stress periods (Bruce et al. 2007). It has been proposed that plants need sufficiently long exposure to drought stress before the appropriate cellular and molecular signals can be generated and then converted into the transcription of stress genes (Trewavas 2005; Vinocur and Altman 2005; Goswami et al. 2010). As an integral component of the developmental plasticity “plant memory” would also impact the genotype-by-environment (GxE) interaction because developmental stage predetermines which genes can be affected in response to stress (De Leonardis et al. 2007). Developmental plasticity and GxE interactions are irreversible. GxE interaction can cause permanent developmental change (West-Eberhard 2003). Thus, optimizing developmental plasticity would benefit the breeding programs to control the GxE interaction.

In this work, we hypothesize that some developmental stages would exhibit higher phenotypic plasticity in wheat. Also, it is highly preferred to detect a phenotypic plasticity during a long developmental stage that needs high water requirements. Because it would be sufficient for building an adaptive mechanism and optimizing drought-tolerant genotypes capture process.

Therefore, we aimed to determine the impact of the main developmental stages on the phenotypic plasticity in response to drought stress; which would minimize the GxE interaction, and optimize the developmental stage to be better suited for capturing drought tolerant genotypes. An ideal stage should satisfy the following criteria: (1) resistance symptoms to drought stress, (2) capable of assessing the tolerance and susceptible responses, (3) high yield fitness, (4) normal germination rate of seeds collected from the drought-stressed plants, (5) normal seedling growth, and (6) withstand the prolonged drought for adaptive response.

## Methods

### Planting conditions

Eight genotypes of spring wheat with different genetic background were evaluated (Table 1). The whole experiment included 480 pots arranged as three replications of eight genotypes in a total of 20 stress regimes (five growth stages by four stress durations) arranged as a completely random design. The experiment was conducted in a walk-in growth chamber (Conviron; Controlled Environments Ltd., Winnipeg, Manitoba, Canada). Plants were grown on a diurnal cycle of 16/8 h at 418 µmol PAR light. Growth chamber temperature was maintained at 22–23 °C/16 °C, and the CO_2_ was approximately 404 ppm of 17% humidity. Seeds were embedded in moist containers, which were filled and compacted with Sunshine Mix LC1 potting soil (Sun Gro Horticulture, Bellevue, WA). An application of 14-14-14 Osmacote (Scotts Miracle-Gro Company, Marysville, OH) slow release fertilizer was incorporated at planting, stress induction, and stress recovery.

**Table 1:** The genotype name, scientific name, ID number, origin, and type of wheat

Genotype	Scientific name	ID No	Origin	Type
Perigee	*T. aestivum*	NASA	NASA-USA	S
Vandal	*T. aestivum*	PI546056	Idaho AES; USDA-ARS	HRS
PWB343	*T. aestivum*	BW26864	India	S
Klein Dragon	*T. aestivum*	CM64693	Argentina	S
PotamS-70	*T. aestivum*	BW623	CIMMYT	S
Indian	*T. aestivum*	CItr4489	USA-Utah	SWS
Onas	*T. aestivum*	CItr6221	South Australia	SWS
Edmore	*T. turgidum var. durum*	CItr17748	USA-North Dakota	DS

*S* spring, *HRS* hard red spring, *SWS* soft white spring, and *DS* durum spring

## Stress regime description and duration

Constant stress was imposed at Zadoks growth stages: 21 for tillering, 45 for booting, 50 for beginning of head emergence (heading) and 60 for beginning of flowering (anthesis) (Zadoks et al. 1974), or not imposed in the case of the control treatment for each of the five growth stage treatments.

All plants were watered uniformly reaching the saturation prior to planting, until the stress imposition was reached for a given pot.

**Fig. 1:**
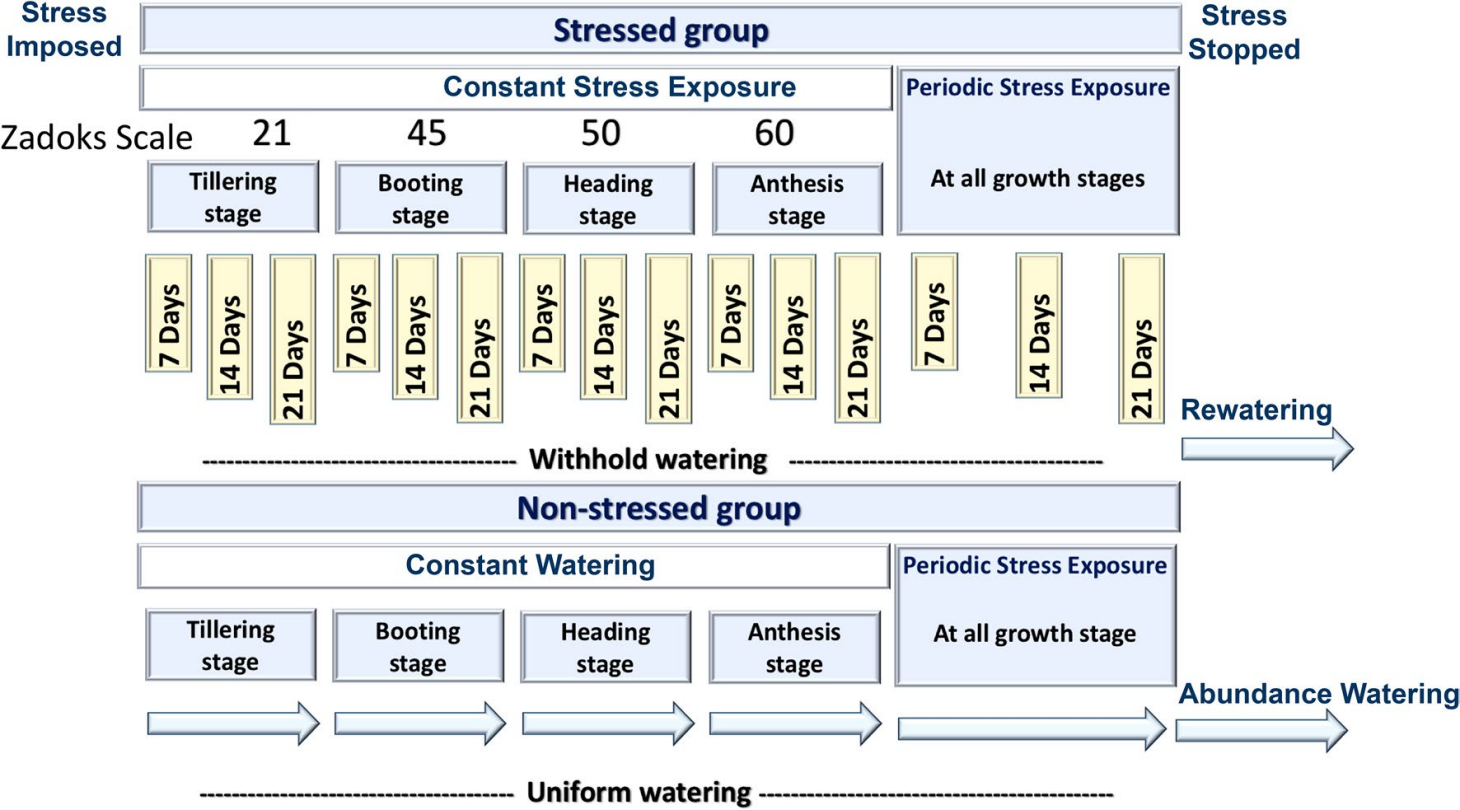
The workflow of the experimental design. It describes the design of the stressed (non-watered) and non-stressed (watered) groups for both of single stress and periodic exposure

The stress was maintained for duration of 0 (control or watered group), 7, 14, and 21 days. For the 7-, 14-, and 21-day treatments, soil saturated was moisture uniformly prior to planting until stress started, then water was withheld for the length of the stress duration, and then plants were re-watered until soil saturation as needed. The 0-day stress treatments were watered throughout the experiment. Stress treatments were also imposed as periodic drought and re-watering cycles. In these periodic stress treatments water was withheld when plants reach a given growth stage for 0, 7, 14, or 21 days, then plants were watered until they reached the next growth stage when stress was initiated again for 0, 7, 14 or 21 days (Fig. 1). The 21-day periodic stress treatments experienced almost constant drought stress from tillering on.

## Measurements

### Phenotypic measurements

The yield component traits plant height (PH), tiller number (TN), total spike number (TSN), grain number (GN), and grain weight (GW) were measured at the end of the experiment for each genotype/stage/stress duration/replicate. All the morphological traits were measured according to Pask et al. (2012).

### Chlorophyll fluorescence

The chlorophyll fluorescence represents the maximum efficiency of PhotosystemII (PSII). The effective photochemical quantum yield of PSII (YII) (Genty et al. 1989; Maxwell et al. 1994; Maxwell and Johnson 2000) was measured in light using, the portable photosynthesis yield analyzer (MINI-PAM-II, Heinz Walz GmbH, Germany). The efficiency of photosystem II was calculated as YII = (F_m_ − F_o_)/F_m_ based on the steady-state photosynthesis under lighting conditions (Genty et al. 1989). where: F_m_: The ground fluorescence of light adapted samples. F_o_: The maximal fluorescence of light adapted samples. YII: The yield of photochemical energy conversion.

The yield of photochemical energy measurement monitors any changes in photosynthetic activity during recovery. The YII was measured at the end of the stress treatment that was imposed at tillering stage for the 21-day duration and it was re-measured ten days after re-watering plants. The corresponding control plants were measured on the same day duration even though they hadn’t experienced stress. Three reads were taken from each fully expanded leaf from each replicate and genotype at the tip, mid and bottom of each wheat leaf, and averaged. The measurements were taken in the light between 12 a.m. and 14 p.m.

### Germination efficiency

After harvest of grain from each plant, ten seeds were chosen randomly from each treated plant (excluding the treatments which failed to produce seeds) to test the seed dormancy. All the grains were synchronized by imbibing with water at 4 °C for 24 h on sterilized CBD3.5 steel blue germination blotters (Ancho paper, St. Paul MN) in Petri dishes covered with aluminum foil and then placed in an incubator at 22 °C. On the 5th day, the germination efficiency was estimated according to Guo et al. (2012) for each plate as follows: *Ge%* = n/N × 100, where *n* is a number of seeds germinated in 5 days; *N* is the total number of seeds.

### Seedling growth

Another set of ten seeds was used for each treated plant (excluding the treatments that failed to produce seeds) to measure the coleoptile length and root lengths of the harvested seeds. The seeds were germinated as above. On the 10th day, both the coleoptile and root lengths were measured according to Pask et al. (2012).

## KASP assay for *Rht* genes

We also studied the relationship between the interaction of the developmental stages and drought stress duration with genes for reduced plant height. The genomic DNA was extracted from leaves from the eight genotypes using the BioSprint DNA Plant Kit (Qiagen, CA, USA) according to the manufacturer’s instructions. The alleles of the *Rht*-*B1* and *Rht*-*D1* genes were assayed using the Kompetitive allele-specific PCR (KASP) protocol. The KASP markers were developed in 2013 part of the MASWheat funding project based on the Rht markers that published previously (Ellis et al. 2002) (http://maswheat.ucdavis.edu/protocols/Dwarf/). The amplification was carried out using the thermal cycler T-100Tm (BioRad Laboratories, Inc.). The assay was genotyped through the endpoint method using the Light Cycler ^®^ 480 instrument II (Roche Diagnostics Ltd. Forrenstrasse, Switzerland). According to the LGC Genomics, Ltd. (Middlesex, UK) guide manual, the reaction components, preparation, and PCR program were performed.

## Statistical analysis

The statistical analyses were done using the SAS-Enterprise and SAS-JMP software. The charts were plotted using GraphPad PRISM version 7.00. The Mean and standard error were calculated, and treatments were compared by an analysis of variance utilizing the SAS GLM procedure with all effects fixed using the model: ${\text{Y}}_{{i{\text{jk}}}} = \upmu + \upalpha_{\text{i}} + \upbeta_{\text{j}} + \upvarepsilon_{\text{ijk}}$ where Y_ijk_ is the grain number (yield) of ijkth observation, µ is the overall mean plus the effects of the (α_i_) ith genotype, the β_i(j)_ is the effect of jth stress duration treatment, nested within the developmental stage, and, ε_ijk_, is the random deviation associated with each observation. The replicate variance was omitted from the model after finding the non-significant variance between the replicates. The LS-means and their 95% confidence intervals for each treatment were calculated from each analysis and were compared using Tukey’s and Dunnett’s comparison tests. The relationships among yield traits were measured using the Pearson correlation coefficient with the pairwise estimation method.

## Results

### Dynamics of plant morphology in response to drought stress

During the constant stress exposure, leaf rolling was the earliest morphological characteristic observed at the end of the 7th day of drought. The next morphological symptom was wilting of leaves that became apparent at 14–21-day of drought. Typically, leaf yellowing was observed by the 14th day of drought followed wilting. Leaf drying was the last leaf symptom before death. Dryness started with brownish spots at the leaf tip appearing by the end of 14th day, which gradually expanded towards the leaf base by the day 21.

**Fig. 2:**
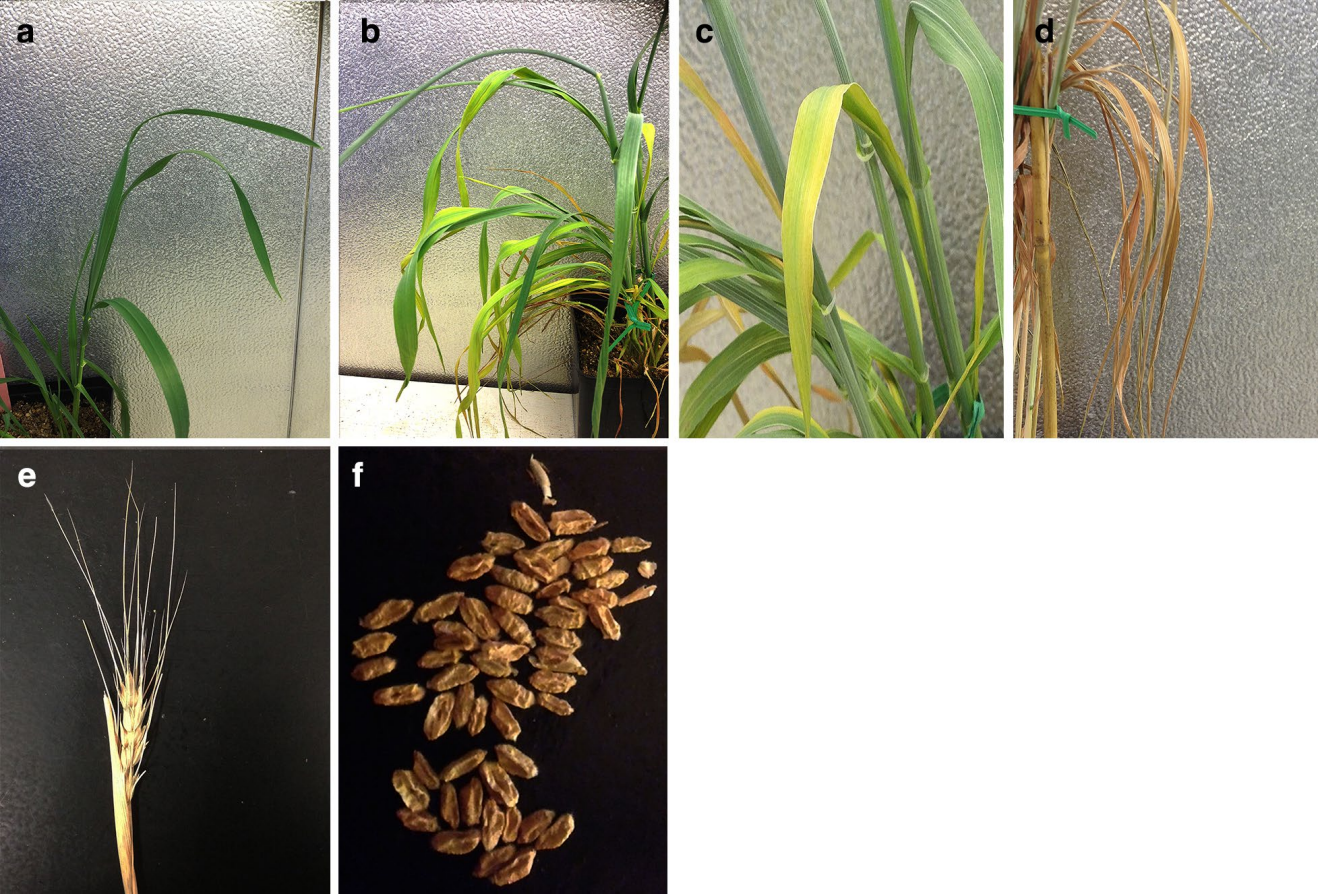
The figure illustrates the development of drought morphological symptoms. It shows the main and unique symptoms were shown during drought exposure. **a** Leaf rolling, **b** Leaf wilting, **c** Leaf yellowing, **d** Leaf dryness, **e** Spike damage (booting stage) and **f** shrunken seeds (anthesis stage)

In the periodic stress exposure, the rolling and wilting recovered after re-watering of 7-day periodic treatment. The chance of recovery was less for the 14-day periodic stress when leaves were yellowing and drying. Surprisingly, plants could survive after 21-days of periodic stress treatment regardless of the developmental stage applied even after all the leaf symptoms (Fig. 2a–d). We observed generation of new leaves in plants after re-watering. In the constant stress treatment, most plants with yellowing and dryness symptoms failed to recover. The genotypes of Klein Dragon, Onas, and Indian were capable of recovering by generating new leaves. The susceptible genotypes such as PotamS-70 could not recover. Although Vandal and Perigee showed the most tolerant symptoms at 7-day treatment but gradually both showed less performance with prolonged drought.

Plants at all developmental stages exhibited dynamics of leaf symptoms described above regarding the stress duration (Fig. 2a–d). Additionally, unique plant and seed morphology were observed at certain developmental stages. When plants exposed to stress at the tillering stage were able to recover faster after re-watering. Leaf dryness at the tip without expanding, and generating new leaves under prolonged drought were observed only at tillering stage. Application of drought during the booting stage resulted in partial or complete inhibition of boots opening for all the genotypes, when awns with only half of the spikelet were seen (Fig. 2e). At the heading stage, we observed decline in the number of spikes, especially that are followed by the main spike. The most severe impact of the drought was at the booting and anthesis stages, causing empty spikes or shrunken seeds (Fig. 2f), especially with the prolonged duration that has obstructed the seed development.

## Evaluating the phenological plasticity

### Yield components and plant height

In this study, the grain number was chosen to represent the yield fitness. There was a high significant correlation (r^2^ = 0.9) between the grain number and grain weight. Other yield components were the number of tillers and the number of spikes, in addition to the plant height. The primary effect of drought was associated with the grain number and the plant height parameters. Through all treatments, a significant reduction (49.19%) in the grain yield occurred between the stressed plants compared with the non-stressed plants.

**Fig. 3:**
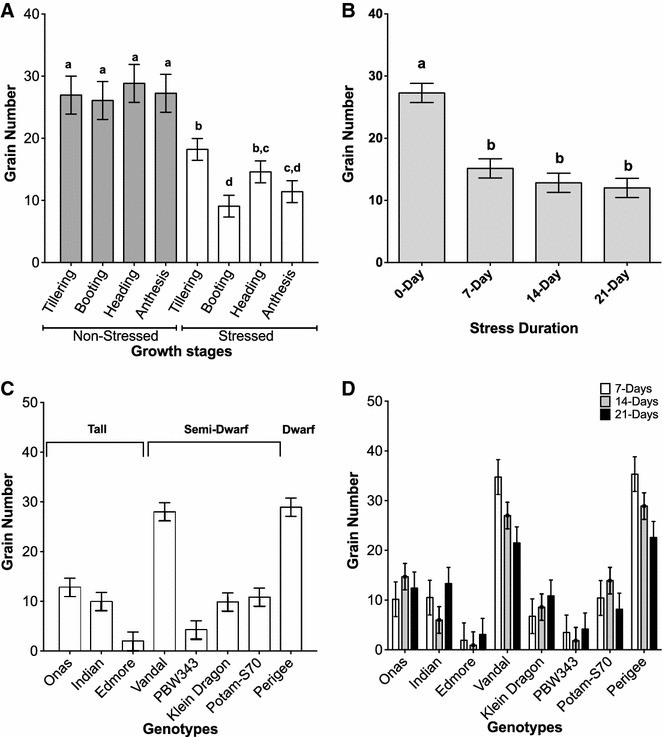
The phenotypic plasticity was measured by the grain number in all charts. The Charts shows that environmental variance of GxE under drought stress was promoted by the adaptive developmental plasticity more than the stress duration only. **A** the developmental plasticity, in respect to the genetic and stress duration variances. An insignificant difference was found between the non-stressed plants and the plants that were exposed to drought stress during the tillering stage. **B** A significant reduction in grain number in response to any of drought stress durations of 7, 14 and 21 days. For **A** and **B**, the displayed letters; *a*, *b* and *c* indicate the significance (P < 0.05; one-way ANOVA and Tukey’s multiple comparisons tests). **C** The distribution of genotype variance in response to drought stress treatments, classified by alleles at the Rht loci. **D** Genotypes demonstrated dynamic responses to prolonged drought. Onas, Indian, Edmore, Klein Dragon and PWB343 had increased grain number for 21 days of drought while others had decreased grain number

An insignificant loss in grain number was found from the plants that were exposed to the drought stress during tillering stage compared with the non-stressed plants. Although there was a similar impact of the drought stress imposed at the heading, anthesis and tillering stages, the grain number from those plants which were drought stress at heading and anthesis stages were different from their control groups (Fig. 3A and Table 2). A significant negative impact was observed at the booting stage in response to drought. At the booting stage, the plant height and total spike number were significantly reduced under drought stress compared with non-stressed groups (Table 3). The total tiller number did not show a significant reduction (Table 3).

**Table 2:** Grain number losses when drought stress was imposed at various developmental stages either as constant or periodic stress, including all the genotypes under 7, 14, 21-days of drought

Stress exposure	Developmental stage	0-day	7-day	14-day	21-day
Constant stress	Tillering stage	26.96 ± 4.02	20.92 ± 4.70	14.33 ± 3.76	19.37 ± 2.82
Constant stress	Booting stage	26.08 ± 3.50	14.67 ± 3.95*	10.17 ± 2.26*	2.42 ± 1.42**
Constant stress	Heading stage	28.83 ± 3.58	12.75 ± 1.79*	16.21 ± 2.72*	14.83 ± 2.52*
Constant stress	Anthesis stage	27.25 ± 3.01	12.21 ± 2.20*	10.62 ± 1.88*	11.42 ± 2.17*
Periodic stress	All the stages	24.29 ± 2.25	6.29 ± 1.31*	3.46 ± 1.90*	9.25 ± 1.90*

* Significant at the P < 0.05 probability by the LSD test compared to the non-stressed group (0-day), **Significant reduction at P < 0.05 compared to the non-stressed groups and other treatments

**Table 3:** Reductions in plant yield components from the non-stress (control) due to drought imposed as constant stress or periodic stress, including the measurements of all the genotypes

Stress duration	Constant stress exposure				Periodic stress exposure			
	0	7	14	21	0	7	14	21
Traits								
Plant height	8.93	0.029	−3.58*	−5.38*	10.8	−4.16	−0.30	−0.36
Tiller number	0.122	0.19	−0.11	−0.21	0.26	−0.16	−0.24	0.14
Total spike number	0.39	−0.027	−0.11	−0.25*	0.54	−0.29*	−0.29*	0.04
Grain number	10.46	−0.168	−3.98*	−4.8*	13.47	−4.53*	−7.36*	−1.57
Grain weight	0.20	−0.03	−0.08*	−0.09*	0.23	−0.032	−0.12*	−0.07

* Significant at the P < 0.05 probability by the LSD test

In consideration of the stress duration and in comparison to the non-stressed groups (0-day), all the stress durations (7-, 14-, and 21-day) reduced the yield fitness of the plants significantly. Overall, in the treatments, the largest reduction in yield fitness occurred between 0 and 7 days of drought (Fig. 3B and Table 2). When the stress was extended to 21 days, the highest reduction in yield occurred when drought was imposed at booting stage. Exclusive to tillering stage, plants that were exposed to stress for 21 days produced higher grain number than the group of 14-days of stress (Table 2).

The genetic effect on the drought tolerance is summarized in Fig. 3C. The grain number was reduced substantially in PWB343 and Edmore and to a lesser extent in Onas, Indian, Klein Dragon, and Potam-S70. Grain number was reduced only slightly in Vandal and Perigee. Based on the results of genotyping, the diagnostic alleles of *Rht* genes, Onas, Edmore and Indian are tall plants (Rht-B1a/Rht-D1a), Vandal, PBW-343, Klein Dragon and PotamS-70 are semi-dwarf (Rht-B1a/Rht-D1b), and Perigee represents a severe dwarf plant (Rht-B1b/Rht-D1b). Exposure to drought for 7 or 14 days had a stronger impact on the yield in the taller genotypes than on the yield of dwarf and semi-dwarf genotypes. However, two tall genotypes Onas and Indian could recover more efficiently after exposure to drought for 21 days than the semi-dwarf genotypes. The tetraploid genotype (Edmore) was more sensitive than Onas and Indian.

**Fig. 4:**
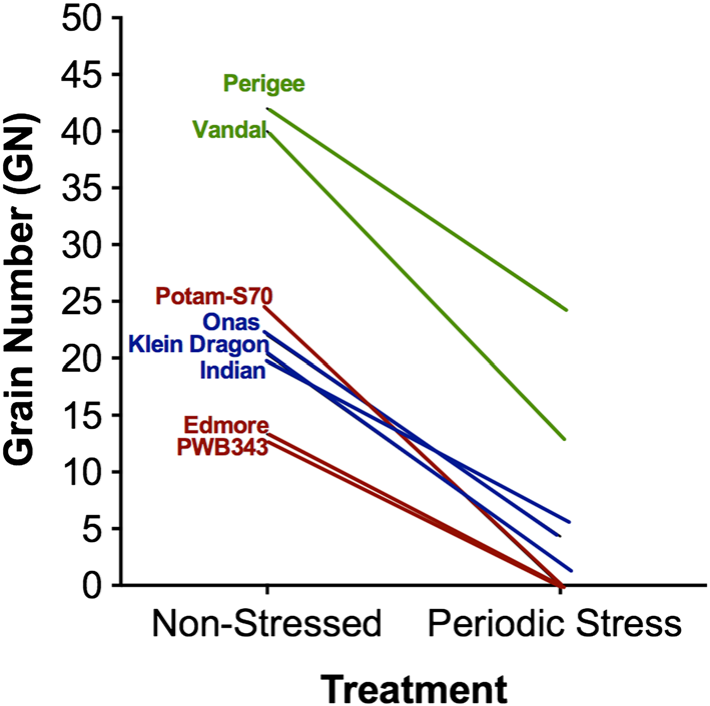
The graph shows the genetic variance in response to the periodic stress. The periodic drought stress has a negative impact on the grain number, and was varied across the different genotyes

In Fig. 3D, the interaction between the genetic variance and the stress duration was examined to identify any adaptive pattern if shown under the prolonged stress. Some genotypes had better grain number from plants with 21 days of stress treatment than with shorter periods (Fig. 3D). Furthermore, periodic stress reduced the yield fitness dramatically (Fig. 4). Grain number of the stressed treatments was significantly reduced compared to the non-stressed treatments (Fig. 4; Table 2). The constant stress exposure resulted in the lowest grain yield (Table 2). In our work, the genotypes showed a varying range of responses to periodic stress (Fig. 4). The grain yield of Vandal and Perigee was reduced less than the other genotypes, with respect to the difference comparing with the non-stressed groups. Onas, Indian and Klein dragon were more unwavering and adaptive. In contrast to constant stress exposure, prolonged periodic stress-induced the adaptive response only in the tall plants (Table 4).

**Table 4:** Means and standard errors for plant height show the impact of the growth stages (tillering, booting, flowering and anthesis), the stress durations (0, 7, 14, 21-days), and the periodic stress in response to drought for specific alleles of the reduced height genes

Description	Tall, Rht-a/a	Semi-dwarf, Rht-a/b	Dwarf, Rht-b/b
*Growth stage effect*			
Tillering-nonstress (control)	70.67 ± 3.05	50.21 ± 2.64	16.33 ± 5.28
Tillering imposed stress	50.63 ± 2.5*	41.01 ± 2.17*	17.28 ± 4.35
Booting-nonstress (control)	70.44 ± 2.03	48.67 ± 1.76	17.67 ± 3.5
Booting imposed stress	32.63 ± 3.0**	30.82 ± 2.6**	13.83 ± 5.2
Heading-nonstress (control)	67.0 ± 2.06	46.83 ± 1.78	16.17 ± 3.57
Heading imposed stress	62.78 ± 1.82	44.42 ± 1.58	12.28 ± 3.16*
Anthesis-nonstress (control)	67.11 ± 1.71	46.33 ± 1.48	14.0 ± 2.9
Anthesis imposed stress	52.15 ± 1.90*	40.25 ± 1.64*	13.05 ± 3.29
*Stress duration effect*			
0-day	68.80 ± 1.11	48.01 ± 0.96	16.04 ± 1.92
7-day	53.28 ± 2.33**	42.36 ± 2.02*	14.0 ± 4.03
14-day	47.55 ± 2.10**	39.22 ± 1.82**	14.87 ± 3.64
21-day	47.80 ± 2.63**	35.79 ± 2.28**	13.46 ± 4.56
*Periodic stress effect*			
Periodic-nonstress control (0 day)	63.77 ± 1.65	44.33 ± 1.43	14.17 ± 2.86
Periodic-7 day	38.89 ± 3.55**	33.08 ± 3.08*	14.17 ± 6.16
Periodic-14 day	44 ± 3.98*	31.92 ± 3.4*	12.67 ± 6.89
Periodic-21 day	47.44 ± 4.4*	28.92 ± 3.81*	9.5 ± 7.6*

* and ** Significant at P < 0.1 and 0.05 level of probability, respectively by the LSD test

In general, the plant height was reduced significantly from the non-stressed control when drought imposed at all developmental stages (Table 3). Our results indicate that plant height was most affected by stress at the booting stage (Table 4). Also, drought inhibited plant height when the stress was applied during the tillering stage. The impact was higher than the heading or anthesis stages showing the greater impact on the tall and semi-dwarf genotypes (Table 4).

In our study, plant height was reduced significantly during tillering, booting and anthesis stages by the tall plants associating with an adaptive response to drought. The height of the semi-dwarf plants reduced significantly but with limited loss in yield at low drought stress conditions. The only dwarf plant (Perigee) that did not show a significant reduction in plant height with booting stage exception. Interestingly, the heading stage has insignificant impact on plant height by the tall and semi-dwarf plants with the only severe dwarf plant exception. All the tall and semi-dwarf plants showed significant reduction of plant height under periodic stress while the reduction was insignificant of the dwarf plant (Perigee) except under 21 days of periodic stress.

### Germination efficiency and seedling development

**Fig. 5:**
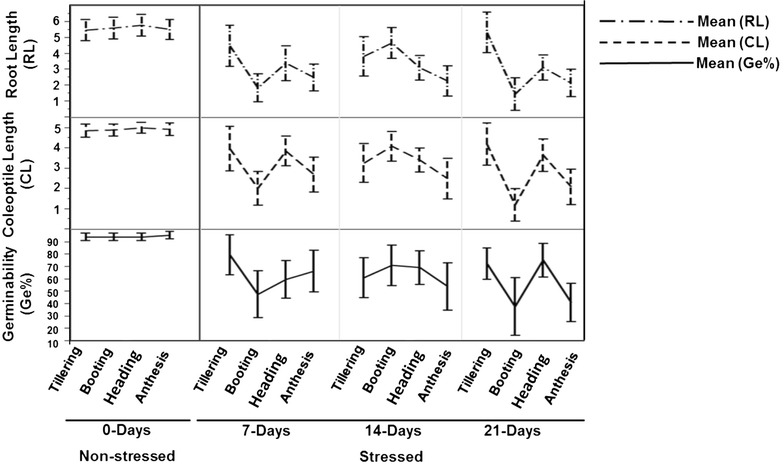
The in vitro germination percentage of the produced seeds. Means and standard errors of in vitro germination percentage for the produced seeds of the used genotypes of spring wheat show the drought response, summarized by the impact of the growth stages for each stress duration

Exposure to drought stress at the anthesis stages for 7, 14, or 21 days of drought resulted in shrunken seeds, with germination efficiency 65 55, and 45%, respectively (Fig. 5). Reduced grain number following drought application at the booting stage was accompanied by an average germination of 53.3%. Though, seeds from plants stressed at tillering and heading stages had a germination percentage of 77, 55% with 7-days; 60, 70% with 14-days and 70, 71% with the 21-days stress, respectively. The length of coleoptiles and roots of 10 days old seedlings were measured to assess the effect of drought on seedling growth.

The coleoptile and root lengths were reduced significantly in the seeds harvested from the stressed treatments (Fig. 5). Across all the developmental stages, the root length correlated significantly with the coleoptile length (r^2^ = 0.83), and the germination percent correlated with the coleoptile and root length (r^2^ = 0.84, 0.82), respectively. Due to the high phenotypic plasticity of tillering and heading stages, the growth slopes of coleoptile length was reduced insignificantly even after drought stress for 21 days. A significant reduction was observed in the slope of the root length decline only at the heading stage, while at the tillering stage was not. The results revealed an association of fitness of grain yield and germination efficiency with seedling growth.

### Analysis of chlorophyll fluorescence and pigments

**Fig. 6:**
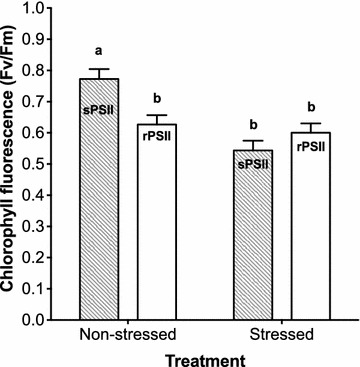
The impact of drought stress on the chlorophyll fluorescence. The photosynthetic activity was measured at the end of the drought stress exposure after 21 days at tillering stage, the PSII compared with non-stressed plants during the stress (sPSII) and after re-watering (rPSII)

The maximum yield of photosystem II was used to assess the photosynthetic activity. Only plants under the adaptive response of the least sensitive stage (tillering stage) were measured at two time points, at the end of 21 days of drought stress (sPSII) and 10 days after re-watering (rPSII). We found a significant reduction of both parameters (Fig. 6). Interestingly, the re-watering period of 10 days had no effect on the rPSII suggesting that optimization for the photosynthesis water deficiency remained stable. The chlorophyll fluorescence of the non-stressed group of the 21 days treatment decreased dramatically from the tillering stage to the re-watering stage. The watering of the non-stressed plants was a scheduled amount until the stressed plants were re-watered. At that time, both non-stressed and stressed treatments were watered to full watering capacity (Fig. 6).

## Discussion

### Tillering stage is a plastic stage for drought screening

The hypothesis was built considering that high plasticity is preferable to be during a long stage that needs high water requirements. In a case of plasticity existence, minimizing this amount of water at long duration would be sufficient for building adaptive mechanism. Touching that topic, some studies stated that wheat would uptake higher portion of the total life water requirement during the vegetative stage, and wheat adapted to less amount of rainfall over years, due to the climate changes (Schillinger et al. 2008). So we assumed that vegetative stage might be carried an adaptive mechanism of water uptake in wheat plants. Because stress duration is an important factor to evaluate the stress severity, we imposed the stress for different drought stress durations (7, 14, and 21 days) at each developmental stages.

As we described in the results, we obtained some dynamic changes in leaf, spike, and seed symptoms, which associated with either a certain developmental stage, stress duration, or genetic response. Apparently, some leaf symptoms were well documented with drought; but, defining the morphological dynamics during the developmental stages or drought regimes are poorly described, although it is important traits and easy to record. These changes in plant morphology under different environments reflect the plastic response (De Jong and Leyser 2012), and are thought to be important to indicate specific physiological changes. Accounting the symptoms with the phenotypic measurements, the plant response pipeline could be drawn. Leaf wilting and rolling are indicated the start of a lack of available moisture in the soil and a consequent shortage of water transport to stem and leaves, then follow by correlation with reduced photosynthesis and pigment content. Also, leaf rolling occurs typically in most grasses including rice, maize, and sorghum during perception of drought induced by increased production of salicylic acid signal (Kadioglu and Terzi 2007). Those symptoms would not be used to indicate the plastic response to drought, because they are common symptoms through the developmental stages, especially if plants would recover after re-watering. As a result of a reduction of the chlorophyll content, leaf yellowing was observed, which can be used as a measurement of the damaging effect of drought (Moore and Lovell 1970). While leaf dryness reflects the cell programmed death. When the plants experienced dryness through the whole leaf under drought stress, this should be a non-recoverable symptom unless there is an adaptive mechanism induced to generate new leaves. Tolerant stressed plants at tillering stage experienced leaf dryness through leaf tip, but through the whole leaf only in the sensitive genotypes. Exclusively, at the tillering stage, the generation of new leaves after a prolonged drought indicated the adaptive response to drought. Booting stage was the least plastic developmental stage, crediting unique negative symptom (inhibition of boots opening) resulted in short spikes, few seeds, empty spikes and low seed vigor. At heading stage, limited regeneration would happen especially after the prolonged drought. The plant would focus to survive the spikes that already were formed. Also, drought stress during the anthesis stage drove the stressed plant to maturation faster, shortening the milk and dough stage resulting in shrunken seeds without generating new leaves. Heading and anthesis stages promoted less correlation between the grain number and grain weight, which might be due to the significant impact of drought on the fertile tillers at the spike formation during the grain-filling. Regarding the productive stages, the perturbation in seed development under drought stress may produce shrunken seeds, which contain low protein and starch content, reducing the germination efficiency and causing abnormal seedling development (Wood et al. 1977; Guo et al. 2012). Fábián et al. (2011) highlighted a greater significant reduction in the size of the mature embryos in the sensitive genotype. In addition, the low germinability was correlated with the size, development and genetic variability of plant population (Li et al. 2014). However, taking credits of high germinability and seedling development would value the phenological plasticity of developmental stages. Also, the association found between germinability of the produced seeds and seedling development effected plant morphology and yield uniquely at different developmental stages indicated the dynamics of developmental plasticity.

Interestingly, although under the prolonged drought, the tillering group recovering to adaptive symptoms were seen. The maximum quantum efficiency (PSII) was reduced by drought. The significant loss of light energy was constantly either during the stress (sPSII) or after re-watering (rPSII). It seems that lowering PSII yield could be energy leakage due to the damaging impact of drought on the chlorophyll (Huang et al. 2009). In the meantime, the constant loss suggesting the possibility of two reasons. First, that optimization for photosynthesis water deficiency remained stable under the adaptive mechanism. Second, abundant watering after constant stress for a long time might be considered as a stress. Fortuitously, we observed a significant reduction of the photochemical efficiency in the non-stressed plants after changing the watering pattern from scheduled watering to water abundant. However, re-watering seems to be a mechanism or perturb mechanism. When plants experience complete recovery following re-watering, the recovery may depend on the pre-drought intensity or duration (Xu and Zhou 2007; Xu et al. 2009, 2010). Unfortunately, scant pieces of literature discussed that phenomenon, especially in cereals. These changes in the phenotypic response caused by the repeated and by prolonged drought may indicate induction of acclimation mechanism, such as desiccation tolerance mechanism (Berjak 2006; Tobias 2012). Periodic exposure to drought stress may alter the developmental program of spring wheat by changing the duration of the individual stages and scaling down the fitness of grain yield.

Exclusively, during tillering stage, plants are capable of recovering after re-watering producing normal seeds and seedling growth. Plants are capable of adapting with less water requirements under long development stage at the prolonged drought compare to the short stress exposure. Under our experimental conditions, we concluded total grain number reduction of 32.46% in tillering stage, 65.18% in booting stage, 49.39% in heading stage, and 58.09% in anthesis stage under the constant stress exposure. While a reduction of 73.92% under the periodic stress exposure. Veesar et al. (2007) reported in *Triticum aestivum* that in order to increase the numbers of spikelet per spike, stress should be avoided at tillering stage. Although they stated under field conditions yield declines of 20.74, 46.85 and 101.23% when drought stress was subjected at tillering, booting and at grain formation respectively. Moreover, Alghabari et al. (2014) stated high sensitivity to drought at booting and anthesis stages were mainly due to grains per spikelet, and mean grain weight, respectively. The tillering stage is taken to give a stable and good yield followed by the heading stage while booting and anthesis had the lowest yield.

## Assessment the resistance to periodic stress is a vital aspect

Moreover, the integrated effect of the periodic exposure to drought through all the stages caused a harsh combination of general and state-specific symptoms. Resistant plants capacity to hold out the physiological and biochemical changes under the periodical drought stress may demonstrate the adaptive response explained by the phenological plasticity. In previous reports, a tolerant genotype produced 18–44% more grain than a susceptible genotype under severe cyclic water limiting conditions due to osmotic adjustment (Izanloo et al. 2008). Similarly, in our research and through the entire experiment, the most tolerant genotype produced 10.6–30.15% grains more than the most susceptible genotype under constant stress exposure and periodic stress exposure, respectively. Which indicates that considering resistance response under periodic drought stress helps to build a robust drought tolerance assessment and capture superior drought genotypes.

### Plant height genes may contribute to the adaptive response

In general, it is documented that changes in plant height is one of the mechanisms for adaptive modulation of plant growth and is mediated by the nuclear transcriptional regulators “DELLA proteins”; which plays the central role in gibberellin (GA) signaling under drought stress. Also, DELLA proteins accumulation restrain the plant growth and increase the capability of survival under drought (Achard and Genschik 2009). After observing a general significant reduction of plant height during tillering, booting and anthesis stages of the tall and semi-dwarf genotypes with a dwarf exception. Meanwhile, tall plants (Onas, Indian and Edmore) have better adaptability only under prolonged drought. We concurred with the previous reports that the impact of height alleles varied with genetic backgrounds and environments, where the tall and semi-dwarf plants exhibited similarly and adaptable to stress under prolonged drought. While the shortest tall wheat genotypes may be useful in marginal drought stress environments (Mathews et al. 2006). Although, Alghabari et al. (2015) suggested that severe dwarf Rht alleles are better able to enhance tolerance to high temperature and drought stress in wheat. Therefore, that observation is worth further studies, especially when the association of plant height’s impact on the developmental plasticity in wheat is not discuss enough in the literature.

## Conclusions

Our results concluded that, (1) the developmental stages accommodate phenotypic plasticity, while the timing factor appeared to be resolving the adaptive plasticity interacting with the developmental stage and genotypes. However, the environmental variance of GxE under drought stress in wheat was promoted by the developmental plasticity. (2) Intense drought stress shifted the phenology of the developmental stages wheat. In depth, tillering stage scored the highest phenological plasticity. Heading stage seemed to be similar to tillering but the low germination and seedling development of produced seeds hindered that plastic response. While, booting and anthesis stages showed a maladaptive response to drought stress. In this study, we recommend considering the resistance response to the periodic drought stress as an important aspect in screening methods. Also, investigating in the impact of plant height alleles on the adaptive response is recommended. We propose tillering stage is to be a promising stage for better suited for capturing drought-tolerant genotypes through a screening method. Resistance to periodic drought stress is an important aspect in screening methods.
